# Novel approaches to smoothing and comparing SELDI TOF spectra

**Published:** 2007-02-20

**Authors:** Sreelatha Meleth, Isam-Eldin Eltoum, Liu Zhu, Denise Oelschlager, Chandrika Piyathilake, David Chhieng, William E. Grizzle

**Affiliations:** a Department of Medicine, Medical Statistics Section,; b Clinical Pathology,; c Pathology Chair Office,; d Nutrition Sciences, Biochemistry & Molecular Biology, University of Alabama at Birmingham, Birmingham, Alabama, USA

**Keywords:** SELDI, biomarkers, denoising, peaks, AUC

## Abstract

**Background:**

Most published literature using SELDI-TOF has used traditional techniques in Spectral Analysis such as Fourier transforms and wavelets for denoising. Most of these publications also compare spectra using their most prominent feature, i.e, peaks or local maximums.

**Methods:**

The maximum intensity value within each window of differentiable m/z values was used to represent the intensity level in that window. We also calculated the ‘Area under the Curve’ (AUC) spanned by each window.

**Results:**

Keeping everything else constant, such as pre-processing of the data and the classifier used, the AUC performed much better as a metric of comparison than the peaks in two out of three data sets. In the third data set both metrics performed equivalently.

**Conclusions:**

This study shows that the feature used to compare spectra can have an impact on the results of a study attempting to identify biomarkers using SELDI TOF data.

## Introduction

The effort to produce an index of all human proteins (the human protein index, HPI) began over twenty years ago. This project pre-dates the human genome project by more than a decade. However, the complexity of the task of creating this index was underestimated and the relative simplicity of the human genome with four known nucleic acids arranged in a linear coding order allowed the process of the sequencing of the human genome to progress exponentially (Anderson et al 2001). The successful completion of the human genome project and the limited correlation between gene expression and protein expression has shifted the focus of much research back to proteins. The emergence of new and improved protein technologies from re-engineered two dimensional (2D) gel systems to Surface Enhanced Laser Desorption/Ionization (SELDI) and Matrix Enhanced Laser Desorption and Ionization (MALDI) time of flight mass spectroscopy (TOF-MS), are all being used more and more in cancer research to aid in the discovery of predictive or prognostic biomarkers. A number of articles in medical journals over the last few years suggest that SELDI TOF-MS spectra can be very effective in correctly identifying and classifying individuals with cancer from those without (Petricoin et al 2002; Rosty et al 2002; Shiwa et al 2003; Xiao et al 2003; Wadsworth et al 2004).

### SELDI Chip (Ciphergen)

The ProteinChip System uses ProteinChip Arrays and SELDI (surface-enhanced laser desorption/ionization) technology for capture, detection and analysis of proteins directly from crude biological samples without labeling or chemical modification. Using a combination of arrays displaying unique chemical-binding properties (e.g. reverse phase, ion exchange, metal ion), subsets of proteins can be affinity captured and purified on the surface of the biochip. The processed arrays containing bound proteins are inserted into the ProteinChip reader, a laser-based time-of-flight mass spectrometer for rapid analysis of and relative quantification of hundreds of analytes in parallel. The ProteinChip array has eight spots. Small areas on each spot on the ProteinChip can be sampled by the laser and activated to emit a spectrum. The height of the spectrum (its y-coordinate) is related to the quantities of proteins of a given size (measured in kilo-daltons – x-coordinate). The y-coordinate thus is a measure of the amount of proteins (similar to the optical density in a 2-D gel spot), whereas the x-coordinate is a constant set of possible values of the molecular weight of the proteins. Each spectrum has between 15000–50000 detectable values. The spectra can either be exported after baseline correction and normalization or as raw unprocessed data. It is also possible to export the spectra with a time scale instead of the m/z scale. They are exported as comma separated files and stored in an excel database.

### Analysis of SELDI Spectra

The ultimate aim of a SELDI experiment is to build statistical models called classifiers to correctly classify individuals into the diseased/non-diseased categories and then to identify proteins that comprise the classifier. A SELDI spectrum has a number of features that can be used to build this classifier. The feature used most often to compare or classify SELDI spectra are the peaks. The maximum intensity in a chosen region and the area under the curve (AUC) that forms a peak are two of multiple features of SELDI spectra that have been used. However, the spectra need to be preprocessed before its features can be used in a classifier. Some of the commonly used pre-processing techniques are described below.

### Normalizing the Spectra

Differential sample loading, differences in surface of spots, fluctuations in laser energy etc, can contribute to variability in the recorded spectra. “Normalization” is the process of subjecting the intensities to arithmetic operations in order to minimize the effect of uncontrollable factors on the intensity. A common way to do this is to divide each intensity value on a spectrum by the total intensity of the individual spectrum. This expresses each intensity value as a percentage of total intensity, and the percentage of total intensity at each m/z value should be the same regardless of factors such as sample loading, chip surface etc.

### Baseline Subtraction

The process of acquiring the spectra can also contribute to differences in intensities in the different spectra due to varying baselines. Baseline subtraction is a pre-processing step, which essentially tries to identify the line (function) that traces the minimum value across the bottom of the spectrum, and then subtracts it from the intensities, so that intensities in all spectra are measured from a zero baseline. Coombes et al 2003 and Baggerly et al 2004 describe a variety of techniques that can be used in baseline subtraction. Ciphergen’s ProteinChip software allows one to use their proprietary algorithms to normalize spectra using a variety of options and subtract baseline from the spectra before exporting it to the database.

### Denoising

Denoising is the process of reducing random noise in a spectrum to make the signal clearer. Fourier transforms (Baggerly et al 2003) and wavelet transforms (Lee et al 2003), are some of the techniques used to denoise spectra.

#### Alignment

The machine reading and recording the intensity at various m/z values is known to have an error rate of approximately 0.2% about its m/z values, i.e. if the data says intensity at m/z 2000 is X, X could represent the intensity at 1,996 or 2004. The spectra from different individuals thus need to be adjusted in some way in order to be sure that the intensities being compared across different spectra represent intensities at the same m/z ratio. Yasui et al (2003) describe a method to align spectra. In this paper we describe a different method of aligning and evaluating spectra.

#### Feature Selection and Dimension Reduction

After pre-processing the spectra, one has to identify the subset of spectral features that will be used to build a classifier. Most publications use two sample tests such as t-tests or Wilcoxon rank sum tests to identify those features (peaks, maximum intensity, or AUCs) that are statistically significantly different between the groups at some preset significance level (e.g. p < 0.0001). These selected features are then used to build a classifier.

### Classifiers

Classifiers are algorithms that are used to divide a group of individuals into different groups. Classifiers that use supervised learning (e.g. Discriminant Analysis (Moreno et al 1995; Weber et al 2002)) are used in a situation where one knows the true groups prior to building the classifier. In this case the features of spectra are used to construct an algorithm that can divide the individuals into the correct groups. In unsupervised classifiers (cluster analysis: Dwek and Alaiya 2003), the aim is to find natural clusters of groups and then investigate the features of the spectra, to understand what they can teach us about the groups.

#### Rationale for this Paper

As mentioned above, Ciphergen’s SELDI machine has an error rate of about 0.2% about its m/z values. Thus the intensity values +/− 0.2% at each m/z values could potentially all represent the total intensity at the same m/z ratio. We thus hypothesized that the area under the curve would be a better measurement of the intensity of a particular protein or multiple proteins/peptides in the sample than the peak or other measure of local maximums such as the maximum intensity in this same region. Further we hypothesized that a classifier that used AUC would have better sensitivity and specificity than a classifier that used local maximums. The paper describes the preprocessing techniques used and compares the quality of these two classifiers. To the best of our knowledge there are no other studies that have compared the effect of feature selection on the quality of the classifier.

## Methods

### Pre-processing

#### Step 1: comparing total ion content

We compared the median total protein intensity in the normal patients, to the median total intensity in the diseased patients using a Wilcoxon Rank Sum test to see if one group of patients is over expressing proteins compared to the other.

#### Step 2: Normalization

Each patient’s intensity values were normalized to his/her total intensity. This is equivalent to normalization by the total ion current. These intensity values were then divided by the sum total of intensities in both groups. Each intensity value is thus expressed as a proportion of the total protein intensity in the experiment. This makes fold change comparisons meaningful.

#### Step 3: Alignment and feature selection

As described above, the machine reading and recording the intensity at various m/z values is known to have an error rate of approximately 0.2% about its m/z values, i.e. if the data indicate an intensity at m/z 2000 is X, X could represent the intensity at 1,996 or 2004 or both. This error rate varies increases with increasing m/z values. Each m/z value was multiplied by 0.002 to create a variable that represented the width of its reliability interval. The distance between adjacent m/z values was calculated by subtracting an m/z value from the one immediately after it. We created reliability intervals, such that the distance between the lowest m/z value in the interval, and the highest m/z value in the interval was less than or equal to the width of the reliability interval for all the m/z values in the interval. All m/z values in an interval were then represented by the highest m/z value of the interval. Thus, all values between 2000 and 2004 were represented by 2004. Table 1 displays this process for the first twelve values in a spectrum. This process was both a process of alignment across the different spectra and a process of data reduction. In this way approximately 1000 m/z values could represent the 10000 or more m/z values in the original spectra. Two features related to these m/z values were used to compare the spectra. The first of these was the maximum intensity value with each window, e.g. 2004 represents values from 2000 to 2004; the largest intensity value within these four intensities is used to represent the intensity at m/z 2004. The second feature was the area under the curve spanned by these four m/z values. The area was calculated using a simple trapezoidal rule; the height of the trapezoid, was the intensity value halfway between the maximum intensity and minimum intensity within this window. The width of the trapezoid was the difference between the largest m/z value in the window and the lowest.

### The Classifier

Wilcoxon Rank Sum test was used to compare the maximum values and AUCs at each m/z value. A subset of the most highly significantly differential peaks (AUCs) was subject to Stepwise Discriminant Analysis (SWD) that was used to select m/z values and intensities to be included in the classifier. Different cutoffs of p-values were used to determine significance in the Wilcoxon Rank sum test for the three different data sets, but when both types of classifiers were used on the same set of data, identical cut-offs were utilized. A random sample of diseased and non-diseased patients was selected. SWD was run on this random sample to identify a subset of the significant local maximums that could classify the patients correctly into disease/non-disease groups.

This process was repeated 10,000 times. In order to avoid over fitting, the number of variables included the classifier was restricted to the a fifth of the study sample (Draper and Smith 1981). The 10,000 lists of candidate m/z values were examined and the most frequently occurring local maximums (AUCs) were included in the final classifier. The covariance matrices in the two groups were tested to see if they were significantly different. If they were, a quadratic function (instead of linear) was used as a discriminating function. The first two data sets were from pilot studies and had very small sample numbers. In this case, a “leave-one-out” cross-validation was used to assess the quality of the classifier. This method randomly selects an individual, leaves him/her out of the sample, calculates the discriminant function using the other individuals and then classifies this “unknown” individual. Cross-validation is understood to yield a fairly unbiased estimate of the sensitivity and specificity of the classifier (Lachenbruch and Goldstein 1979). The third data set was large enough to allow one to separate the cases and controls into training and test data sets. In this case the quality of the classifier was determined by the quality of classification in the test set.

## Data Sets Used

The first pilot data set consisted of SELDI profiles of sera from twenty-one women with normal pap smears and twenty-one women with abnormal (HSIL) pap smears. The purpose of the study was to see if the spectra of the two groups were different enough to enable one to separate the two groups out.

In the second data set body cavity fluids were obtained from the University of Alabama at Birmingham. Twenty-two samples consisted of 10 pleural fluids obtained by thoracocentesis, 7 pelvic washings obtained during hysterectomy and/or oophorec-tomy, 4 peritoneal fluids by paracentesis, and 1 peri-cardial fluid by pericardiocentesis. The aim of the study was to determine whether protein profiles generated by SELDI-TOF MS could differentiate reactive/inflammatory conditions from neoplastic disease.

Qu et al (2002) have described the third data set used in this study. Specimens from two groups of patients were used in this study: 80 age-matched controls and 80 patients diagnosed with organ-confined PC A (T1/T2). A donor was selected for the control group if he had a normal digital rectal examination (DRE), a prostate specific antigen (PSA) level of less than 4.0 ng/ml, and no evidence of prostatic disease. The control group consisted of 40 Caucasian and 40 African-American males ranging in age from 51–70 years (mean age, 60 years). The organ-confined PCA group (T1/T2) consisted of 76 Caucasians, 20 African Americans, 1 Asian, and 2 men of unknown race with ages ranging from 50–89 years (mean age, 71 years).

## Results

### Data Set 1: HSIL versus Normal

The first data set consisted of SELDI spectra obtained from the sera of 21 women with normal pap smears and of 21 women with HSIL. Figure 1a shows the averaged spectra in the Normal and HSIL. The spectra are fairly similar. In Figure 1b is a closer view of the spectra between 7200 m/z and 9600 m/z. Figure 1c displays the same region (7200m/z to 9600 m/z) of the original spectrum, and the aligned, smoothed spectrum. This figure suggests that the alignment and feature selection using maximums, works to reduce the dimensionality of the data to one tenth of the original data (10458 m/z values to 1074), but keeps the main characteristics of the original spectrum. Figure 2a represents the trace of the maximum intensities between 7000m/z and 10000m/z region of the spectrum. Figure 2b is the trace of the AUCs between 7000m/z and 10,000m/z. As one can see, the AUC trace also provided a fairly good summary of the original spectrum, although the magnitude of the AUCs are as expected much larger than the values of just the maximums at a particular m/z value. These findings were repeated in the other two data sets used in the study.

#### Total Protein Content

The total protein content in each sample was estimated by adding up all the protein intensities in an individuals’ spectrum. Wilcoxon’s rank sum test was used to see if the total protein content in the two groups were significantly different. In this data set total protein content in the two groups was not significantly different (p = 0.77). Thirteen m/z values had maximums that were significantly different at p = 0.05.

The quadratic discriminant classifier that used maximums had a cross-validated specificity of 76% and sensitivity of 62%. Thirty-three AUCs were significantly different at p = 0.05. The classifier that used AUC instead of peaks had 100% specificity and 67% sensitivity.

### Data Set 2

This data set had SELDI spectra from the pleural fluid of eight patients with various diagnoses of cancer and fourteen patients who had normal pleural or peritoneal biopsies. The total protein content in the fluid of the cancer group was significantly higher (p = 0.0044) than the protein content in the fluid from the normal group. 84 m/z values had significantly different maximum intensities at p = 0.0002. The quadratic discriminant classifier that used the local maximums had a cross-validated specificity = 100% and sensitivity = 62.5%. 39 m/z values had significantly different AUCs at p = 0.0002. The quadratic discriminant classifier that used AUCs had a cross-validated specificity = 100% and sensitivity = 100%.

### Data Set 3

The third data set is one that has been extensively analyzed and discussed in the various publications (Qu et al 2002; Diamandis 2003; Yasui et al 2003).

We used SELDI spectra from 80 normal cases and 88 prostate cancer cases (stage a or b) obtained from the Microbiology and Molecular Cell Biology laboratory at the East Virginia Medical School. The total protein content in the two groups was significantly different (p < 0.0001) but in this case it was higher in the normal cases, when compared to the cancer cases. For this analysis, we adapted the macro used previously to select subsets of cases and controls before the Wilcoxon rank sum test to identify maximums or AUCs that were significantly different. Sixty-eight cases and sixty controls were randomly selected to form the data set. The remaining twenty cases and twenty controls were set aside as the test set. Forty cases and controls from each group were then selected from the training set, at each run to identify significantly different intensities or AUCs. These values were then subjected to SWD, and lists of potential m/zs were stored. The process in this case was replicated 5000 times. The quadratic discriminant classifier using local maximums had a test set specificity of 90% and sensitivity = 95%. The classifier using the AUC fared a little worse, with specificity = 90% and sensitivity = 85%. Both classifiers had seven variables. Five of the seven m/z values identified by the two techniques were identical.

## Conclusion

The protocol described in this paper is unique in several ways. To the best of our knowledge, the technique used to align the m/z values has not been described elsewhere.

The method reduces the dimensionality of the data, while keeping its main characteristics, and we believe has the added advantage of being intuitive and thus easily replicated. We are also not aware of any other study that has compared the total protein content in the two groups being studied. The results here suggest that total protein content may be able to distinguish between cancer and non-cancer specimens; this is very important in that analysis without the correction for total protein will always separate cancer from non-cancer in such a group. The biological bias and/or plausibility of these findings are under evaluation in our laboratory. We are planning additional experiments to evaluate these issues. In this analysis, the maximum intensity or AUC at every set of differentiable m/z values is being tested for significant differences. The advantage of AUC versus peak intensity, or the local maximum used in this study, is not yet clear. The results from the first two studies seem to suggest that AUC may be more sensitive than using local maximums. However this conclusion was not confirmed by the third study, which had a larger sample size and used a test data set for assessing accuracy of the classifier.

## Figures and Tables

**Figure 1a:**
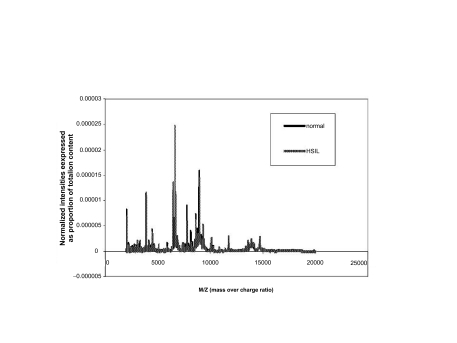
Normalised mean spectra.

**Figure 1b:**
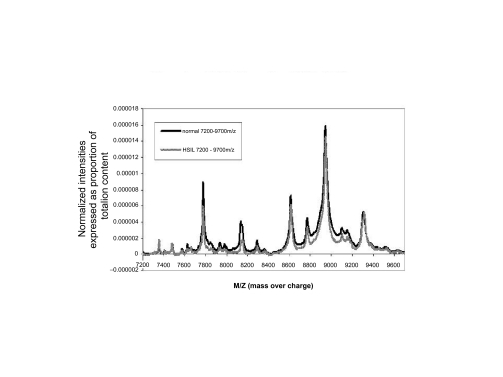
Normal and HSIL Normalized 7200—9700.

**Figure 1c:**
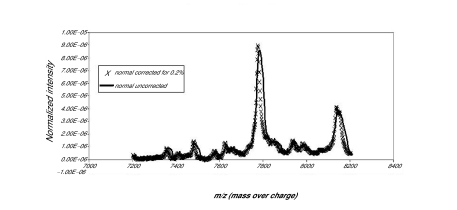
Normal mean and maximum s.

**Figure 2a:**
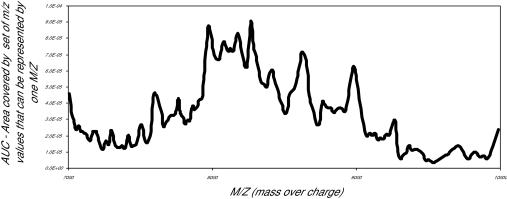
Normal AUCs 7000—10000 M/Z.

**Figure 2b:**
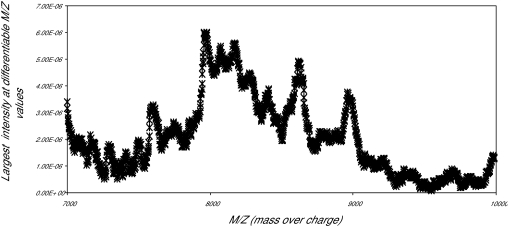
Maximum values m_z 7000—9000.

**Table 1:** Aligning spectra and adjusting for error in measurement. Bolded italicized and underlined values in column 1 indicate the beginning of a new window of m/z values that are all represented by the new m/z value in column 2. The new window starts at the m/z value at which the cumulative sum of the distances between consecutive m/z values (column 5) is greater that the reliability interval in column 3. Column 4 is the distance of each m/z value from its immediately preceding m/z.

M/Z	M/Z NEW	Reliability Interval = m/z *0.0 02 (rounded)	Distance from last	Cumulative Distance
2000.2475	2003.5541	4	0	0
2001.0739	2003.5541	4	0.8264	0.8264
2001.9004	2003.5541	4	0.8265	1.6529
2002.7272	2003.5541	4	0.8268	2.4797
2003.5541	2003.5541	4	0.8269	3.3066
***2004.3811***	**2007.6911**	***4***	***0.827***	***4.1336 (set to 0)***
2005.2084	2007.6911	4	0.8273	1.6547
2006.0358	2007.6911	4	0.8274	2.4823
2007.6911	2007.6911	4	0.8277	3.31
***2008.519***	***2011.8324***	***4***	***0.8279***	***4.1379 (set to 0)***
2009.3471	2011.8324	4	0.8281	0.8281
2010.1754	2011.8234	4	0.8283	1.6564
2011.0038	2011.8234	4	0.8284	2.4848
2011.8234	2011.8234	4	0.8286	3.3134
***2012.6612***	***2015.978***	***4***	***0.8294***	***4.1428(set to 0)***
